# Effect of the addition of silica obtained from rice husk on physicochemical and mechanical properties of fibercement

**DOI:** 10.1016/j.heliyon.2023.e13567

**Published:** 2023-02-09

**Authors:** D.M. Gomez Mejia, D.F. Hincapie-Rojas, F.N. Jimenez-Garcia, César Augusto Alvarez Vargas

**Affiliations:** a Departamento de Física y Matemáticas, Universidad Autónoma de Manizales, Colombia; b Departamento de Física y Química, Universidad Nacional de Colombia Sede Manizales, Colombia; c Tecnoparque nodo Pereira, Sena Regional Risaralda, Colombia; d Departamento de Mecánica y Producción, Universidad Autónoma de Manizales, Colombia

**Keywords:** Rice husk, Rice husk ash, Silica microparticles, Fibercement, Mechanical properties, Physicochemical properties

## Abstract

After replacing asbestos with other types of fibers used as reinforcement of cementitious matrices, it has been found that rice husk, an agro-industrial waste with high silica content, can be used to improve the properties of fibercement. In this work, the effect of adding different forms of silica (rice husk, rice husk ash, and silica microparticles) on fibercement's physicochemical and mechanical properties was investigated. Rice husk ash and silica microparticles were extracted from the rice husk incineration and acid leaching process. The chemical composition of silica was determined by X-Ray Fluorescence, and the ash leached with hydrochloric acid was found to contain more than 98% silica. Cement, fiberglass, additives, and different forms of silica were used to manufacture fibercement specimens in their different forms. Concentrations of 0%, 3%, 5%, and 7% were taken for each form of silica, and four replicates were performed. The setting time was 28 days, during which absorption, density, and humidity tests were performed. Experiments were statistically analyzed at a 95% confidence value, and it was determined that there are significant differences in the compressive resistance, density, and absorption in relation to the type of additive and the interaction between the type of additive and its percentage of addition, but not whit percentage of addition. It was found that the fibercement specimens with 3% of rice husk present a modulus of elasticity of 9.4% higher than de control sample. The use of rice husk as an additive in fibercement composites seems to be interesting because these agro-industrial wastes are inexpensive and easily available everywhere to utilize in the cement industry and also helpful in reducing environmental pollution due to their cost and the positive effect on their properties.

## 1 Introduction

Fibercement is a material made from cement, fibers, additives and water, characterized by being light and resistant, which is used in construction, especially in lightweight systems, for coating, insulation, and waterproofing [1]. Previously, some cement-based materials made in the Hatschek process by dewatering of dilute suspension of cement–fiber mixture were reinforced with asbestos fibers. However, it became established that asbestos causes incurable illnesses such as pulmonary asbestosis, mesothelioma, and poor-prognosis lung cancer after a latent period of 20–50 years [2,3]. For this reason, it had to be eliminated, which meant the implementation of other alternative materials such as slag mineral wools, rice husk (RH), amorphous silica, and refractory, organic, synthetic, glass [4] and acrylic fibers [5,6]. Law 1968 of 2019, banned the use of asbestos across the national territory of Colombia since 2021. For this reason, the replacement of this material introduces fibercement in the construction area as a new material with characteristics of resistance and elasticity interesting for builders, engineers, and architects. The incorporation of natural fibers and other alternative materials such as industrial pozzolans and fly ash [7] opens the door to the use of silica. This mineral is made up of silicon and oxygen found in nature and can also be extracted from agro-industrial waste such as rice husk to be used as a raw material in construction [8].

Rice is one of the three most consumed kinds of cereal as whole grain. Its husk is a hard layer that protects the grain and is composed of amorphous silica; due to its high silica content, it is used as organic matter to incorporate into the soil because of its low nutrient content; in addition, its physical-chemical constitution is challenging to biodegrade [9].

Rice husk and rice husk ash (RHA) have several applications in catalysis, as pozzolanic materials for soil stabilization, as an adsorbent for waste water treatment, and as an adsorbent for air pollution, among others [10]. There RHA can be used in the production of biodiesel, the pyrolysis of RHA produces biochar that has been used to catalyze transesterification and esterification reactions [11]. This subproduct of the rice industry is also used in construction materials, mortars and concrete, rigid materials that have low tensile strength and low ductility, and which, because they contain cellulose, hemicellulose, and lignin, reinforce construction systems [4]. In addition, their incorporation in cement helps to reduce the use of reagents that generate CO_2_ in its production [12].

FAO's estimate of world rice production in 2021 now stands at 517.1 million tons, an increase of 0.7% over the record level of 2020 [13]. At the national level, the rice harvested area grew from 518 thousand hectares in 2019 to 580 thousand in 2020, i.e., more than 12% [14]. RH constitutes about 20% of the total volume of the grain and contains a large amount of silica. Unfortunately, a large part is unsuccessfully incinerated or thrown into water sources, which harms the ecosystem. This problem has interested some researchers, Serrano et al. [15], who analyzed the possibility of using RH with and without pretreatment as an addition in the manufacture of lightweight mortars. They found that mortars of very low density and high porosity are obtained, making them candidates to elaborate construction materials for thermal and acoustic insulation. Behel et al. [16], evaluate fresh (workability) and mechanical properties of concrete with 0%, 5%, 10%, 15%, and 20% of rice husk ash (RHA) to replace Ordinary Portland Cement. They obtained satisfactory results that augur an excellent performance of this material as a pozzolanic additive. The Compressive strength, split tensile and flexural strength are increased with an addition of up to 15% of RHA. Moron et al. [17], analyzed the properties of lime and cement mortars made with recycled ceramic aggregates reinforced with three types of fibers: glass, basalt, and carbon. It was found that the addition of fibers improves the material's mechanical properties and decreases its shrinkage, which favors the incorporation of recycled aggregates in the manufacture of mortars. Mohammed et al. [18] investigated the mechanical behavior and ductility of ultra-high performance fiber reinforced concrete containing a high volume of glass microfibers (GFM). It was concluded that a lower binder water ratio results in better mechanical performance. In addition, mixtures containing 1.5%–3% glass microfibers yielded higher compressive strength reaching 160 MPa.

After reviewing the literature, it has been found that there is not enough research oriented on the study of fibercement, but rather the study of concrete and cement. The results show that the different pozzolanic materials offer higher resistance values to cement and concrete, their mixture with reinforcement fibers is still an area of exploration. For this reason, more studies are required to focus on the effect of the addition of different forms of silica on fibercement reinforced with glass fibers.

This work aims to study the effect of the addition of silica in different forms (ground rice husk, rice husk ash, and silica microparticles) and proportions (0%, 3%, 5%, and 7%) on the physical and mechanical properties of fibercement composites. The first phase corresponds to obtaining silica from RH and characterizing the raw material. The second phase involved the production of fibercement samples and the study of their physicochemical and mechanical properties. The physicochemical properties analyzed were humidity, absorption, and density; and the mechanical properties were compression and flexural strength. Morphological, thermal, and structural properties were also analyzed according to the type and proportion of silica used. This study is carried out to take advantage of this agro-industrial waste as an additive and natural silica source in cementitious matrices.

## 2 Materials and methods

The procedure followed to obtain the silica and manufacture the fibercement boards is described below. In addition, each of the characterization steps carried out on the silica and the fibercement boards and the statistical analyses carried out with the results obtained from the design of experiments are described.

### 2.1 Obtaining and characterization of silica in its different forms

Rice husk ash and silica powder were obtained following the methodology proposed by Salavati-Niasari et al., [19]. RH was obtained from cultivation in the central zone of Colombia (Tolima). It was washed with tap water to remove impurities and contaminants and dried in a forced convection oven at 100 °C for 1 h. Then it was then taken to an electric mill to reduce its average particle size. The RH was burned in a muffle to eliminate the organic materials and reduce the carbonaceous materials to obtain ash. The ash obtained was purified by leaching with hydrochloric acid, for which HCl solutions were prepared at 37% J.T Baker at concentrations of 1 M, 2 M, and 3 M, and added to 5 g of rice husk ash; the solution was then brought to a temperature of 85 °C on a plate with magnetic agitation. The resulting paste was washed five times with distilled water, filtered using a vacuum pump, and checked for neutral pH [[20], [21], [22], [23], [24]]. To obtain the silica microparticles, a dry silica sample was taken and crushed in the mortar, then passed through a certified sieve with a diameter of 8″ (203.2 mm), a height of 2″ (50.8 mm), and a mesh size of 90 μm, to ensure a more uniform particle size. A semi-quantitative analysis was performed on the micro-silica obtained using an X-ray fluorescence spectrometer, MagixPro PW-2440 Philips with SemiQ5 software by 11 scans to detect the elements present in the sample excluding H, C, Li, Be, B, B, N, O, and transuranic elements.

### 2.2 Processing of fibercement specimens

Fibercement specimens were made with a mass of 300 g, Cemex Vertuá plus hydraulic cement type MM/B(P–C)-28 plus silica in different forms 80.2%. Calcium carbonate (marmoline CaCO_3_ 90%) 14.70%. Glass fiber Cem-FIL 60 filament diameter: 14 μm and length of 12 mm 3.10%. Bentonite 1.60%, and high-range water-reducing plasticizer based on polycarboxylates of very high fluidity and water reduction from Sumiglas S.A 0.4%. A water/cement ratio of 0.3 was used, and other concentrations of the different forms of silica 0%, 3%, 5%, and 7% were taken based on previous work [25]. For each formulation (see Table 1), the design of experiments was performed, with four replicates, for a total of 40 samples for the compression test, and 40 samples for the flexure test, the samples were randomly selected for the analyses. The fibercement components were mixed according to ASTM standard C305–20, 2020 [26].

**Table 1:** The formulation for fibercement with a total mass of 300 g.

Samples	Cement (g)	Calcium carbonate (g)	Water (mL)	Plasticizer (mL)	Fiberglass (g)	Bentonite (g)	Sílice form (%) (g)	
MC	240.60	43.80	165.0	1.20	9.30	4.80	0	0.000
MCA-3	233.38	43.80	165.0	1.20	9.30	4.80	3	7.220
MCA-5	228.57	43.80	165.0	1.20	9.30	4.80	5	12.03
MCA-7	223.76	43.80	165.0	1.20	9.30	4.80	7	16.84
MCE-3	233.38	43.80	165.0	1.20	9.30	4.80	3	7.220
MCE-5	228.57	43.80	165.0	1.20	9.30	4.80	5	12.03
MCE-7	223.76	43.80	165.0	1.20	9.30	4.80	7	16.84
MM-3	233.38	43.80	165.0	1.20	9.30	4.80	3	7.220
MM-5	228.57	43.80	165.0	1.20	9.30	4.80	5	12.03
MM-7	223.76	43.80	165.0	1.20	9.30	4.80	7	16.84

*MC = Control sample, MCA-3, MCA-5, MCA-7 sample rice husk at 3%, 5%, and 7%, MCE-3, MCE-5, MCE-7, sample with ash at 3%, 5%, and 7%, MM-3, MM-5, MM-7, sample with micro-silica at 3%, 5%, and 7%.

All of the above components were accurately mixed following the experimental design presented in Table 1. The raw materials were placed in an aluminum mixing vessel and mixed for 5 min with an electric mixer. The mixture was stirred at a frequency of 13 at 0 rpm and room temperature until homogeneity and moldability were achieved, according to ASTM Standards ASTM C109/109 M −16a, 2016 [27]. The specimens were compacted in a triple plastic mold with a 2" (50.8 mm) rim. The ASTM Standards ASTM C78/C78 M − 21, 2010 [28] was employed for the preparation of specimens subjected to the flexural test. Then, the specimens were compacted in a prism shape with dimensions of 150 mm × 50 mm × 10 mm. Finally, the samples were left to set for 28 days at room temperature and with a relative humidity of 70±10%.

### 2.3 Thermal characterization of fibercement specimens

Thermogravimetric analysis was performed to evaluate the thermal stability of the samples MC, MCA, MCE, and MM at 3, 5, and 7 %wt., using Themys ONE equipment with 10 °C/min heating ramp, under an N_2_ atmosphere from room temperature until 700 °C. This analysis was carried out to determine the organic decomposition of material, the percentage of weight loss due to decomposition, and dehydration, depending on the type of additive and its percentage of addition.

### 2.4 Structural and morphological characterization of fibercement specimens

For structural characterization, the X-ray diffraction technique was used to identify the phases present in the MC, MCA, MCE, and MM samples at 3, 5, and 7 %wt. The samples were pulverized and sieved to obtain a homogeneous powder. Diffraction patterns were obtained using a RIGAKU, MINIFLEX II diffractometer operated at room temperature, equipped with a Cu Kα radiation source (λ = 1.540562 Å), X-ray source at 30 kV, and 15 mA; the scanning was performed between 5° and 70°, in 2θ scale and with a step of 0.02°/s. For morphological characterization, scanning electron microscopy (SEM, Quanta-250, FEI) was employed after preparing the samples with gold coating, with an accelerating voltage of 17 kV. The chemical composition (semi-quantitative) of the sample was obtained through X-ray energy dispersive spectroscopy (EDS) (GENESIS APEX2i).

### 2.5 Mechanical characterization of fibercement specimens

A compression test was performed to measure the compressive strength of fibercement composites according to ASTM C-109 [27]. The measurements were performed on a UNITED uniaxial universal testing machine, with a maximum load capacity of 100 kN. Height and contact area measurements were taken for the cubes using a caliper gauge with ±0.05 mm resolution to calculate Young's modulus from the slope of the linear part of the stress-strain graph within the elastic limit.

The three-point bending test consisted of applying load to three points to cause bending of the material, according to the standard ASTM C78-02, Standard Test Method for Flexural Strength of Concrete [28].

### 2.6 Physico-chemical characterization of fibercement specimens

Relative density and water absorption measurements were performed according to ASTM Standards C 830-00 [27]. The samples resulting from the bending were cut into pieces of 50 mm × 50 mm × 10 mm, then were taken to a forced convection oven at 90 °C for 12 h, then they were subjected to saturation in water for 24 h. The relative density and water absorption were calculated from the wet weight, apparent weight, and dry weight using equations (1.1), (1.2), respectively. Moisture measurements were taken from the samples resulting from the compression test. The moisture percentage was determined on a moisture analyzer balance.

(1.1) $$\rho =\frac{dry\;mass}{wet\;mass-underwater\;mass}*water\;density$$

(1.2) $$\%\;water\;absorption=\frac{wet\;mass-dry\;mass}{dry\;mass}*100\%$$

### 2.7 Statistical analysis of the variables established for the experimentation with fibercement sheets

Statistical analysis was performed to correlate the results of the independent and dependent variables considering in the manufacture of the fibercement samples. The independent variables were: i) variation of mixing percentage at three levels 3%, 5%, and 7 %wt.; ii) mixing material at three-factor levels: rice husk, rice husk ash, and silica microparticles. A randomized, replicated (1 original + 3 replicates) single block experiment was conducted. The following response variables were determined: modulus of elasticity, modulus of rupture, relative density, water absorption, and moisture. A multifactorial analysis of variance of multiple response variables (MANOVA) with 95% confidence and maximum second-order interactions between factors was performed for each selected-response variable. All ANOVAs were verified to comply with the assumptions of normality of residuals, homoscedasticity of residuals, and data independence. A Fisher's LSD test with 95% confidence was applied to determine the groups of different means for each response variable.

## 3 Results and discussion

### 3.1 Chemical composition of silica

**Fig. 1:**
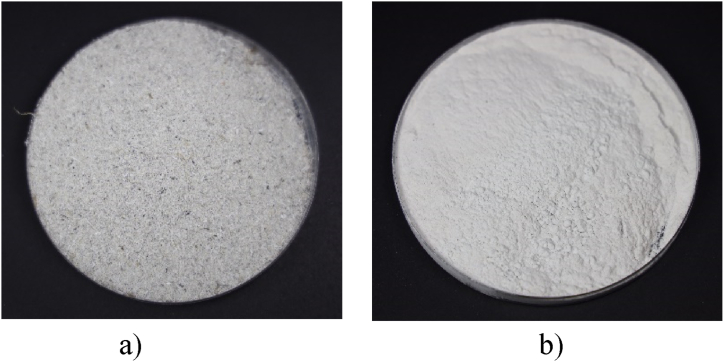
a) Rice husk ash without chemical process, b) rice husk as rich in silica.

HCl-based acid solutions were used at different concentrations to remove impurities in the RHA. Fig. 1a shows the rice husk ash, which presents black impurities, and Fig. 1b shows the silica microparticles in which the removal of impurities has been carried out and presents a white color indicative of higher purity.

**Fig. 2:**
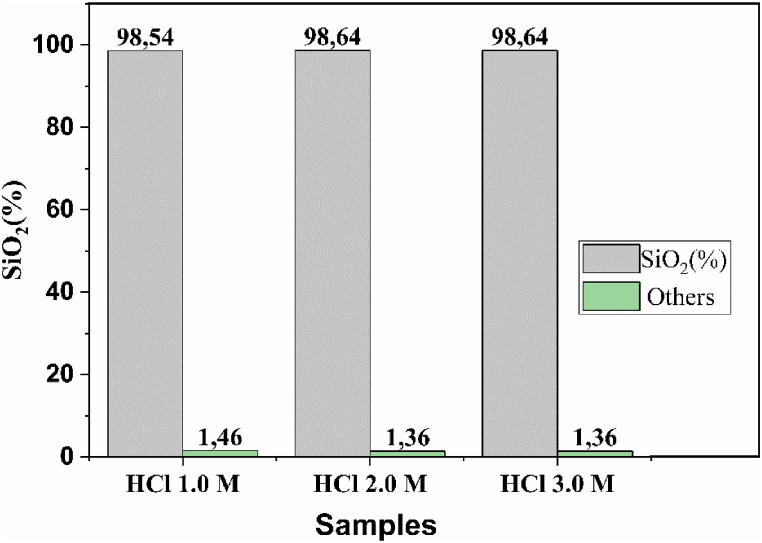
Comparison of silica purity with different concentrations of HCl.

According to literature suggestions, HCl was used for the acid leaching process [28,29]. This acid presents higher effectiveness in removing inorganic impurities such as oxides versus other acids such as H_2_SO_4_, NHO_3_, and HP0_3_ [[30], [31], [32]]. The bar chart in Fig. 2 presents the results of ash leaching for the three concentrations of HCl (1 M, 2 M, 3 M). The ash sample leached with 1 M HCl solution presented a silica content of 98.54%, and the remaining percentage, i.e., 1.46%, corresponds to impurities such as potassium, calcium, magnesium, manganese, aluminum, iron, chlorine, sulfur, phosphorus, sodium, lead, zinc and rubidium. No significant changes in the silica microparticle composition were evidenced by using different concentrations of hydrochloric acid. For 2 M and 3 M concentrations, the silica content present in the ash was 98.64%; therefore, it is considered sufficient to use a 1 M solution for the acid leaching process to minimize costs and waste.

### 3.2 Rice husk and silica microparticles morphology

**Fig. 3:**
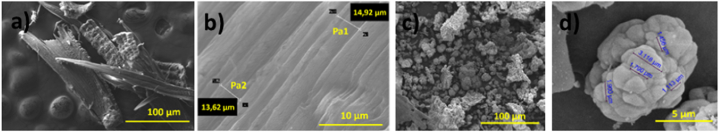
(a, b) SEM micrographs of rice husk, (c, d) SEM micrographs of silica.

Fig. 3a–d shows the micrographs of RH and silica microparticles taken by Scanning Electron Microscopy. Fig. 3a shows the abaxial or external surface of the rice husk, called the exocarp, which is characterized by having a symmetrical structure constituted by convex cells (presence of simple papillae) [4], which are separated by grooves and grains of silicon compounds dispersed over the entire surface (Fig. 3b). Two measurements were taken for the grooves corresponding to 13.63 μm and 14.92 μm, it can be seen in Fig. 3b.

Fig. 3c shows the structure of the micro-silica particles that present irregular shapes of different sizes and in the form of agglomerates; and Fig. 3d shows a magnification of the sample in which agglomerates of silica microparticles tend to have a spherical shape and are made up of individual particles measuring between 1 and 4 μm approximately.

### 3.3 Thermal characterization of fibercement specimens

**Fig. 4:**
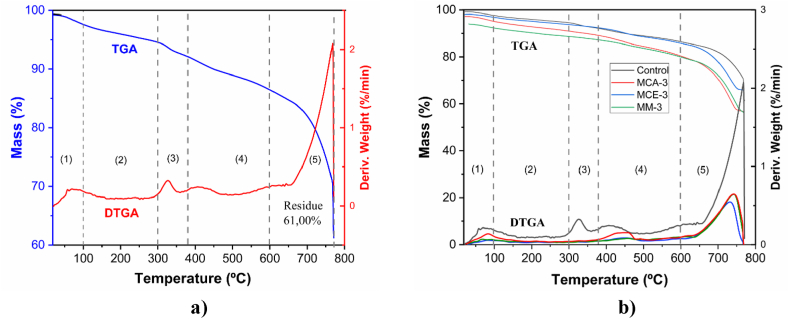
Thermal behavior of fibercement samples with different silica additions, a) control sample, b) samples MC, MCA-3, MCE-3, and MM-3.

TGA analyses were performed for samples MC and MCA-3, MCE-3, MM-3, MM-5, and MM-7. The thermogram in Fig. 4a, corresponding to the control sample, shows five zones associated with fibercement decomposition, which can be identified from the DTGA derivative curve (in red). The first zone is identified from room temperature to 300 °C, with two contributions. The first one for temperatures below 100 °C the mass loss corresponds to the evaporation of water physically adsorbed in the porosity of the sample, and water bound by surface tension, hence the event initial corresponds to the humidity of the sample. In comparison, the second event between 100 °C and 300 °C is related to dehydration processes of the hydrated phases of fibercement such as tobermorite gel, ettringite aluminates, and hydrated calcium aluminosilicates, among others [32,33]. The second event between 300 °C and 370 °C, is associated with the decomposition of polymeric fibers such as cellulose, hemicellulose, and lignin present in the RH [25] and the polycarboxylate plasticizer used in the present work [35]. Between 400 °C and 600 °C, dehydroxylation of portlandite occurs [36], and the last zone between 600 °C and 800 °C is associated with CaCO_3_ decarboxylation [37].

Fig. 4b shows the thermograms of fibercement samples (MC, MCA-3, MC3-3, MM-3). An identical behavior zones mentioned above are observed. The main behavior of the fibercement is predominant in which dehydration, portlandite decomposition, and decarbonization are evident [34]. Table 2 shows the mass loss values for each zone, and it is evident that the highest mass loss occurs in the decarbonization zone.

**Table 2:** Mass loss in the different zones of the thermogram.

	Evaporation of absorbed water (%)	Cement components dehydration (%)	Polymeric fibers and polycarboxylate plasticizer decomposition (%)	Portlandite dehydroxylation (%)	CaCO_3_ decarbonization (%)	Residue (%)
**MC**	2.40	2.84	2.43	5.68	25.66	61.00
**MCA-3**	4.90	4.15	1.62	8.87	24.07	56.39
**MCE-3**	3.21	3.06	1.26	6.69	20.07	65.72
**MM-3**	7.70	3.57	1.32	7.40	18.56	61.45
**MM-5**	2.35	6.12	4.99	6.63	24.89	55.02
**MM-7**	1.40	4.69	3.68	6.88	34.50	48.85

A higher percentage is present in the decarbonization zone due to the decomposition of calcium carbonate into calcium oxide (CaO) and carbon dioxide (CO_2_) during firing [36]. In zone 4 (dehydroxylation of portlandite) and zone 5 (decarbonization of CaCO_3_), the highest percentages of mass loss stand out, indicating a more significant presence of hydrated phases typical of fibercement. The sample with the lowest residue corresponds to the fibercement with the addition of 3% husk (56.39% residue). This fact is because the sample contains a higher content of organic material concerning the other formulations chosen for the analysis. The residues of the other samples are above 61%, which corresponds to the inorganic components, i.e., the minerals present in the ash. The zone in the thermogram with the lowest mass loss corresponds to the decomposition of polymeric fibers and polycarboxylate plasticizer, possibly because these components were used in a smaller proportion in the formulation to make the fibercement specimens.

Table 3 shows the portlandite percentage in the samples. Regarding the amount of portlandite, the highest percentage was found in samples MM-5 and MM-7, and the lowest content in samples MCA-3 y MCE-3. Portlandite has positive effects on the matrix as it is responsible for maintaining the pH of the paste at high values (12–13) by acting as an alkaline reserve, thus keeping reinforced cement-based materials protected against electrochemical corrosion. It is also positive in the case of concretes and mortars with limestone aggregates since, in them, the aggregate-paste adhesion is more significant than in the case of siliceous aggregates. However, it also has adverse effects, since it is soluble in water and easily leached by dissolution; it can react with sulfates and crystallize in dihydrate form, giving rise to expansion and rupture processes and subsequently to the formation of ettringite; it is also the first material to decompose at high temperatures (600 °C). Several of these negative aspects can be controlled with the addition of pozzolans (fly ash, rice husk ash, silica fume, furnace slag, among others) [38].

**Table 3:** Content of portlandite.

Sample of fibercement	Loss of mass	Portlandite (%)
**MC**	4.91	1.19
**MCA 3%**	4.62	1.12
**MCE 3%**	4.20	1.02
**MM 3%**	5.70	1.39
**MM 5%**	6.63	1.61
**MM 7%**	6.88	1.67

### 3.4 Structural characterization of fibercement specimens

**Fig. 5:**
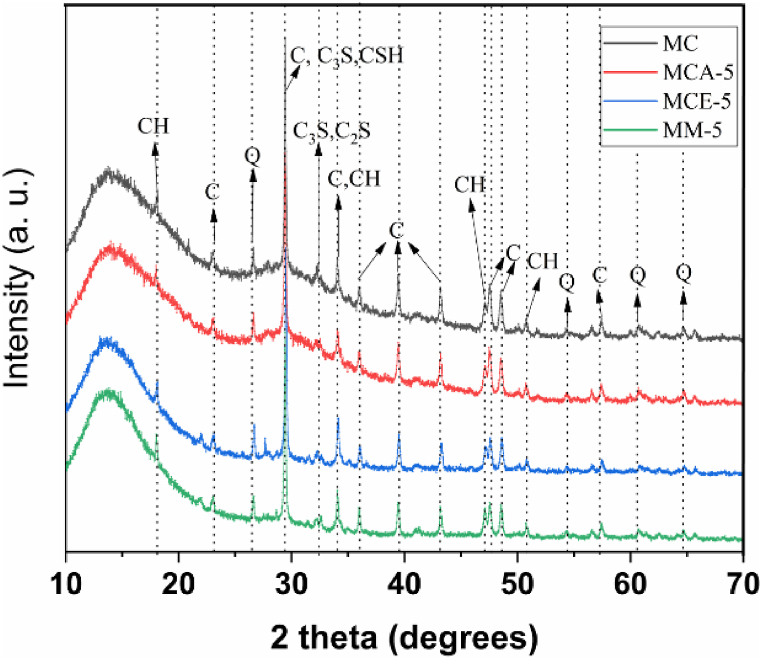
Diffractogram of fibercement samples MC, MC-5, MCE-3, MM-5.

Fig. 5 shows the X-ray diffraction analysis of samples MC and MCA-5, MCE-5, and MM-5. It should be noted that fibercement is a complex material that contains multiple phases, and its identification is not a simple process. However, some phases, such as portlandite (CH: Ca(OH)_2_) with peaks at 2θ = 18°, 34°, 47°, and 51°, are comparable with the powder diffraction datasheet (PDF # 44–1481), tricalcium silicate (C_3_S: 3CaO·SiO_2_) (PDF # 31–0301) at 2θ = 39°, 32°, and dicalcium silicate (C_2_S: 2CaO·SiO_2_) (PDF # 33–0302) at 2θ = 32°, as well as those associated with calcite (CaCO_3_) at 2θ = 23°, 29°, 34°, 39°, 42° and 57° (PDF # 01-072-1937), quartz (SiO_2_) phase (PDF No. 01-085-0798) 2θ = 26°, 54°, 60°, and 66°, tobermorite (CSH: Ca_5_Si_6_O_16_(OH)_2_·4H_2_O) at 2θ = 29° [39] are present in the diffraction patterns of all samples. The presence of these phases is in agreement with the results of the TGA analysis. It should be noted that the main hydration products in cement are tobermorite gel (CSH) and calcium hydroxide (CH), which are formed through cement hydration.

### 3.5 Morphological analysis of fibercement specimens

**Fig. 6:**
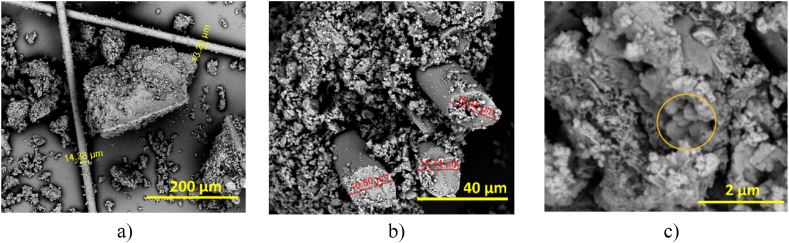
SEM images for the fibercement control sample: a) Size of glass fibers b) Particle adhesion c) Stacking of hydration products. The components identified by EDX for this sample are silicon, oxygen, calcium, aluminum, iron, silicon, and calcium, which correspond to the hydrated phases identified in the XRD analysis.

SEM analyzes were performed on the control sample to identify its morphology and the main hydration phases of the fibercement. The MC-3 sample was also analyzed to contrast the morphological changes caused by the addition of RH. Fig. 6a shows the morphology of the fibercement (control sample) in which the glass fibers are observed with diameters that coincide with the data from the CEMFIL-60 datasheet of 14 μm. Fig. 6b shows particles corresponding to the additives used in the formulation and their adhesion to the fibers, and Fig. 6c shows the stacking of the hydration products. As mentioned above, the adhesion of the additives to the fibers is responsible for the compressive strength.

**Fig. 7:**
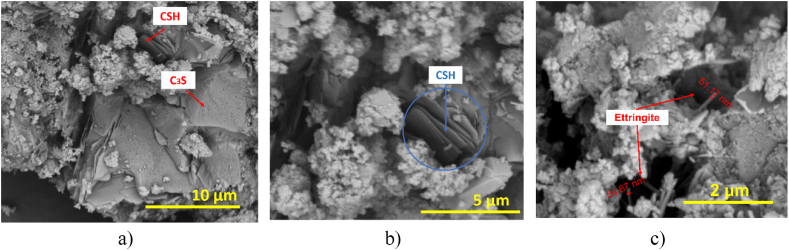
SEM analysis for MCA-3 fibercement: a) 10 μm scale tobermorite gel crystals b) 5 μm scale tobermorite gel crystals c) ettringite.

Fig. 7a shows the morphology of the MCA-3, showing the stacking of tobermorite gel crystals (CSH) and other structures such as the elite (C3S), products that favor the strength of the material [25]. In Fig. 7b, a magnification is made for tobermorite gel crystals, and in Fig. 7c, hydration products such as ettringite, which is needle-shaped, are evidenced. These fibrous forms have a diameter of 14.67 nm and 51.17 nm; these structures are found in empty spaces and fissures that generate negative aspects in the cement since they affect durability and strength, due to the expansion caused inside the matrix.

A chemical analysis EDX was carried out for sample MCA-3, and the presence of representative peaks of oxygen, iron, aluminum, silicon, and calcium was evidenced. These elements correspond to the formation of aluminosilicates and ferrosilicates, cement components identified in XRD. It has been reported that the formation of hydrated calcium silicates (CSH) formed from the reaction of CH (pozzolanic reaction) can be higher in fiber-cement slabs in which silica has been added than in the slab without additions. This case is being presented with the addition of silica and is evident in the SEM images for the sample with 3% silica compared to the control sample.

### 3.6 Statistical analysis of the variables established for the experimentation with the fibercement test specimens

Considering the factors controlled for the manufacture of the fibercement samples: mix percentage and kind of additive, the results obtained in the randomized experiment in which the response variables obtained were elastic modulus, rupture modulus, density, and absorption are presented. The p-values obtained from the ANOVAs are presented in Table 4, and the results for each response variable are described.

**Table 4:** ANOVA.

	Elastic modulus	Rupture modulus	Density	Absorption
Source of variation	**p-value**	**p-value**	**p-value**	**p-value**
**Main effects**				
A: Kind of additive	0.0360^a^	0.4223	0.0036^a^	0.0149^a^
B: Mix percentage	0.5773	0.4178	0.5283	0.0847
**Interactions**				
AB	0.0222^a^	0.4093	0.0407^a^	0.0187^a^
LSD standardized error	31.350 MPa	0.720 MPa	0.015 $\frac{g}{cm^{3}}$	0.227
Maximum coefficient of variation (%)	10.50	7.51	0.90	1.26

a These p-values imply significant effects.

#### 3.6.1 Mechanical property analysis

**Fig. 8:**
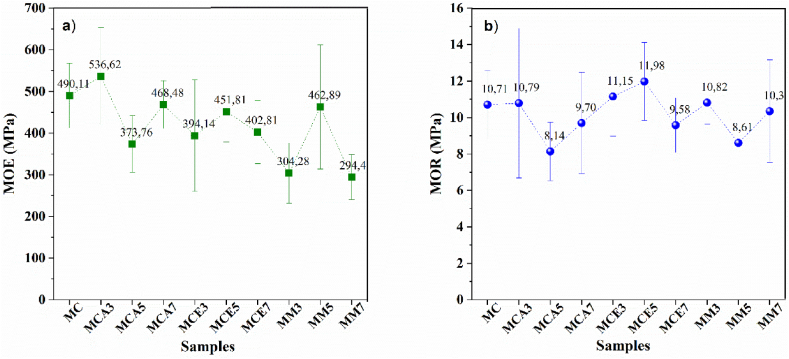
Mechanical characterization of fibercement as a function of the type of additive and its percentage of addition: a) Modulus of elasticity–MOE, b) Modulus of rupture-MOR.

Fig. 8a shows the modulus of elasticity (MOE) for the different samples. The sample MCA-3 presents the highest values for the elastic modulus (average of 563.62 MPa), which increases by 9.4% concerning the control sample (average of 490.11 MPa). An inspection of the MOE values for the other samples indicates that in specimens to which RHA and silica microparticles have been added, there is a decrease in the modulus of elasticity at selected concentrations. The p-values show statistically significant differences (p less than 0.05) in the elastic modulus about the type of additive and the combination between type and percentage of additive, but not for the percentage of additive. According to standard NTC 4373 (Civil Engineering And Architecture. Fibre -Cement Flat Sheets), the modulus of elasticity is between 2000 MPa and 4000 MPa. However, we worked with cubic specimens, prepared according to standard NTC 5069 (Prefabricated fibercement panels. Compression test), for which values were obtained between 294.48 MPa and 536.62 MPa.

Fig. 8b shows the flexural strength of the specimens under study. Values between 7 MPa and 14 MPa were found, consistent with those reported by NTC 4373 (Civil engineering and architecture, Fibercement flat slabs category three modulus of rupture), which determines a range between 9 MPa and 11 MPa [40]. A higher value is evident for the samples MCE-3; however, there are no significant differences in the maximum flexural strength with any of these variables (type of additive, percentage of additive, or a combination of both). This fact can be associated with the amount of glass fiber, which controls this behavior [18], and because the same percentage was used for all the samples in this experiment, no significant differences were found.

**Fig. 9:**
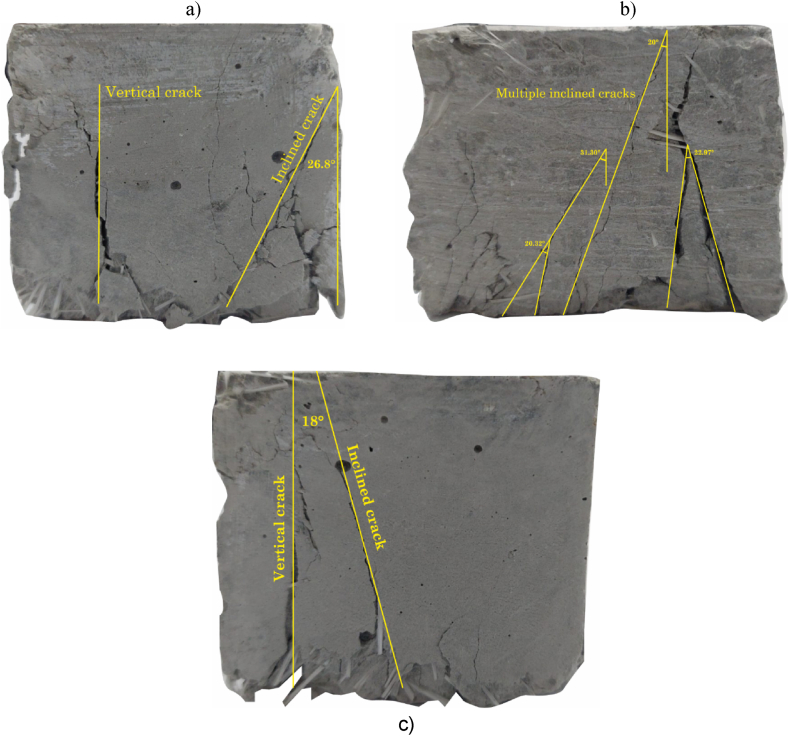
Fracture surface analysis of the broken samples, a) MC, b) MC3 and c) MM5.

Additionally, the fracture mode of the specimens in the fracture surface analysis of the broken samples is shown in Fig. 9a–c. Fig. 9a shows the fracture mode of sample MC, a typical vertical compression crushing crack. Presence of typical inclined lateral crack in brittle compression. Fig. 9b shows the fracture mode of sample MC3 where multiple sloped cracks are identified. The inclination angles are similar showing the homogeneous influence of shear forces to produce brittle failure. Finally, the fracture mode of sample MM5 is shown in Fig. 9c, a typical vertical compression crush crack. Presence of typical inclined lateral crack in brittle compression.

#### 3.6.2 Absorption, density, and moisture analyses

**Fig. 10:**
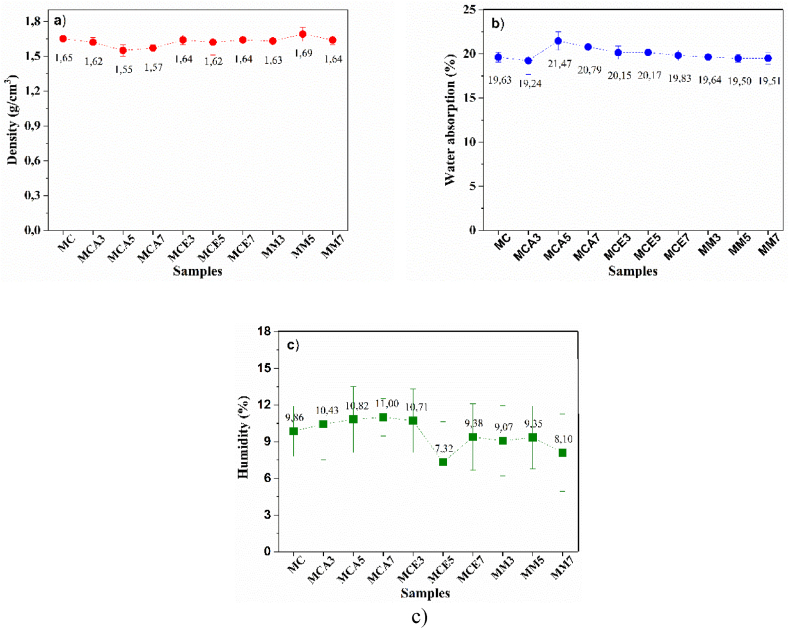
Physical properties of fibercement: a) relative density, b) water absorption, c) humidity.

Fig. 10a–c shows the results obtained for the relative density, water absorption, and humidity of the samples; physical properties are calculated as explained in the methodology section. Fig. 10a shows very homogeneous measurements for density around 1.62 g/cm^3^, values that agree with the reports and technical data sheets for fibercement (i.e. 1.625 g/cm^3^, 1.31 g/cm^3^, and 1.25 g/cm^3^ [40]). The density in cement is close to 3.15 g/cm^3^ and decreases depending on the amount of additive used. Therefore, when fibercement specimens are made, the density should be lower than that of concrete because additives such as those used in this work are incorporated: calcium carbonate, bentonite, glass fiber, and silica.

Fig. 10b shows the absorption percentages of the samples, which are around 20%, a result that agrees with those reported in the literature: 18%, 33%, and 30% [40].

#### 3.6.3 Interactions between additive and percentage of addition

**Fig. 11:**
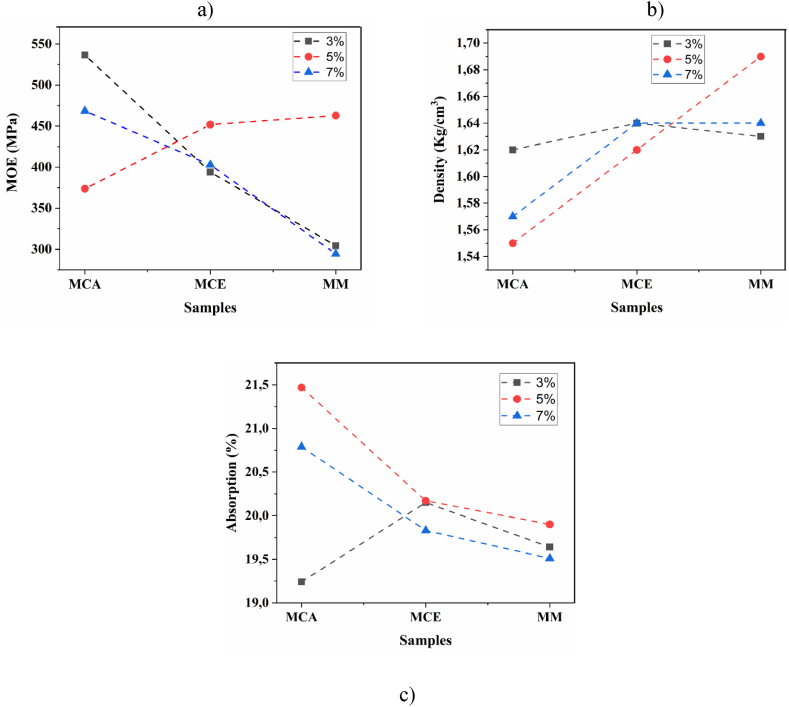
Interactions between additive type and additive percentage for: a) MOE, b) density, c) adsorption.

Fig. 11a-c shows the comparison between the response of the modulus of elasticity, density, and water absorption concerning the interaction between the percentage of addition and the form of silica. Fig. 11a shows that the highest compressive strength values were obtained with rice husk at a mixture percentage of 3%.

Fig. 11b shows that the lowest density value is obtained with RH at 5%, while at the same mixing ratio, the density is maximized for micro-silica. The difference in density values can explain these results for rice husk (0.7 g/cm^3^), ash (0,89 g/cm^3^), and micro-silica (2.2 g/cm^3^); which when incorporated into the cement, modify the final density of the mixture. Fig. 11c shows that the interaction between the factors allows verifying the inverse relationship between density and absorption for the samples where rusk was added. For MCA-3, the density is higher than MCA-5 or MCA-7; besides, it has lower absorption, and at the same time, presents higher compressive strength. This fact can be explained in that the addition of silica acts to improve the microstructure and as an activator to promote the pozzolanic reaction with calcium hydroxide (CH), which leads to a more significant accumulation and precipitation of hydrated products in the available open pores that were initially filled with water. These phenomena lead to the formation of a homogeneous microstructure that can generate higher compressive strength; this type of process has been reported for the addition of silica particles in fibercement slabs [16].

It was reported an increase of 6% in the elastic modulus of cement mortars when RH husk is added at 5%, and an increase in water absorption; values which are comparable to those obtained in this work for fibercement specimens. The improvement in compressive strength is associated with increased adhesion of the cement mortar and the RH and increased friction and adhesion between them. This same author reports a decrease in the elastic modulus for higher values of RH addition (1%–25%), a result comparable to the present work for fibercement specimens in which the elastic modulus decreases when 5% and 7% of RH is added. Tunji et al., [41] reported that the elastic modulus of the cement specimens with rice husk addition at 1.5%, 2.5%, 5.0%, 7.5%, and 10% also showed a decrease; however, the value at 1.5% is like that of the control specimen. The decrease in the compressive strength of the specimens with increasing RH content is attributed to the lower hydration reaction in the mortar as a result of the predominance of rice husk in the composite material, which generates crosslinking between the fibers and result in reduced cohesion between the mortar and the husk.

Finally, the compressive strength values between the 3% rice husk sample and the control sample are compared. A qualitative single factor ANOVA with 95% confidence was performed to determine if there was a significant difference between the factor levels. The ANOVA complies with the normality of the residuals and independence. Homoscedasticity of residuals was tested with Levene's test with 95% confidence; the p-value of 0.039 indicates that the residuals are homoscedastic. The ANOVA test is then validated, and it is specified that there is a significant difference between the control specimen and the specimen with 3% rice husk as far as compressive strength is concerned. According to [47], concrete mixed with rice husk ash as a replacement for cement and glass fibers as an additional reinforcement element present higher compressive strength for an ash content of 15%, which decreases as the ash content increases. The authors indicate that this decrease in strength with increasing ash replacement above the optimum content is due to the lack of hydration.

## 4 Conclusions

In this work, different forms of silica and addition percentages in fibercement were studied to determine the material's mechanical response. The aim was to add value to rice husks as agro-industrial waste by establishing the characteristics of their use in this application. The following conclusions were reached.

The phases identified by XRD for both the control and other samples, such as portlandite, tricalcium silicate, dicalcium silicate, calcite, and tobermorite, correlate with the elements found in the EDX analysis (silicon, oxygen, calcium, aluminum, iron). They are the basis for the identification of the structures identified by SEM.

The physicochemical variables of the samples in this study agree with literature reports: fibercement density was around 1.62 g/cm^3^, absorption was around 20% and humidity averaged 10%. An inverse relationship between density and absorption was observed, which is understandable since a lower density implies greater porosity in the material and therefore there are more spaces for water absorption. This behavior was more noticeable for the mixture with rice husk since it is thicker than the cement particles, which favors water absorption.

According to the results of this work, the fibercement specimens with the addition of rice husk at 3% present higher values in the modulus of elasticity, which may be due to the presence of products that favor the resistance of the material, such as piles of crystal plates of portlandite tobermorite gel, and alite, in addition to hydration products such as ettringite. In addition, the adhesion generated between the rice husk and the cementitious matrix also favors its mechanical resistance.

The improvement in the compressive strength of the fibercement specimens by incorporating 3% rice husk is an interesting result for a possible industrial scale-up since its obtaining compared to that of the other forms (ash and micro-silica) is energetically and economically more favorable since it does not require thermal or chemical treatments.

TGA analysis identified some areas of interest where the dehydration of typical hydration products in fibercement, such as tobermorite, ettringite, and portlandite, occurs. Additionally, the decarbonization of calcite at temperatures above 600 °C was also observed. Some of these hydration products were identified in the morphological analysis of the material.

## Author contribution statement

Diana Marcela Gomez Mejía, Daniel Fernando Hincapié Rojas, Francy Nelly Jimenez García, Cesar Augusto Alvarez Vargas: Conceived and designed the experiments; Performed the experiments; Analyzed and interpreted the data; Contributed reagents, materials, analysis tools or data; Wrote the paper.

## Funding statement

This work was supported by the Universidad Autónoma de Manizales [project 072–179].

## Data availability statement

No data was used for the research described in the article.

## Declaration of interest's statement

The authors declare no conflict of interest.
